# Subclinical and Asymptomatic Atrial Fibrillation: Current Evidence and Unsolved Questions in Clinical Practice

**DOI:** 10.3390/medicina55080497

**Published:** 2019-08-18

**Authors:** Andrea Ballatore, Mario Matta, Andrea Saglietto, Paolo Desalvo, Pier Paolo Bocchino, Fiorenzo Gaita, Gaetano Maria De Ferrari, Matteo Anselmino

**Affiliations:** 1 Division of Cardiology, “Città della Salute e della Scienza di Torino” Hospital, Department of Medical Sciences, University of Turin, 10126 Turin, Italy; 2 Division of Cardiology, Electrophysiology Lab, Sant’Andrea Hospital, 13100 Vercelli, Italy; 3 Cardiology Department, Clinica Pinna Pintor, 10129 Turin, Italy

**Keywords:** subclinical atrial fibrillation, stroke, ischemic cerebral events, catheter ablation, screening, cognitive impairment

## Abstract

Atrial Fibrillation (AF) may be diagnosed due to symptoms, or it may be found as an incidental electrocardiogram (ECG) finding, or by implanted devices recordings in asymptomatic patients. While anticoagulation, according to individual risk profile, has proven definitely beneficial in terms of prognosis, rhythm control strategies only demonstrated consistent benefits in terms of quality of life. In fact, evidence collected by observational data showed significant benefits in terms of mortality, stroke incidence, and prevention of cognitive impairment for patients referred to AF catheter ablation compared to those medically treated, however randomized trials failed to confirm such results. The aims of this review are to summarize current evidence regarding the treatment specifically of subclinical and asymptomatic AF, to discuss potential benefits of rhythm control therapy, and to highlight unclear areas.

## 1. Introduction

Atrial fibrillation (AF), the most common sustained arrhythmia [1], is associated with an increased risk of thromboembolic events such as transient ischemic attack (TIA), ischemic stroke with overt neurological sequelae [2], or micro-embolic events resulting in subclinical brain lesions (revealed by neuroimaging techniques). On the other hand, AF is independently associated with a higher risk of developing dementia [3], with up to a 30% increased risk regardless of clinical cerebrovascular events [4]. Therefore, given the high prevalence of AF in the general population and its considerable impact on both life expectancy and quality of life, correct and prompt management of the arrhythmia is mandatory. However, early diagnosis can be difficult in the case of an asymptomatic presentation, defined as sustained AF episodes in patients not presenting palpitations, dyspnea, fatigue, or other AF related symptoms [5]. The exact percentage of asymptomatic presentations among patients with AF has not been clearly established. Different studies provide estimates between 10% and 40%, according to the characteristics of the population in exam [6,7,8,9,10]. A higher prevalence has been reported in patients with persistent AF, males, elderlies, and in the presence of relevant comorbidities [7]. However, subclinical AF can also be diagnosed in patients with fewer risk factors [10] as paroxysmal AF, for example, at the early phase of arrhythmia development and progression. Moreover, variability in terminology among studies, as later discussed, increases uncertainty related to the description of asymptomatic AF.

In the present review, we shed light on several aspects of this clinical entity, highlighting doubtful elements for which further research is needed: first, we discuss the complex pathophysiological links between AF, dementia, and stroke, focusing on the clinical impact of asymptomatic AF, which has been demonstrated not to be a benign condition. Subsequently, we address the issue of screening for AF, which is of great interest from both a clinical and population medicine point of view. Finally, we discuss management of the patient with asymptomatic and subclinical AF, considering clinical recommendations, anticoagulation, antiarrhythmic drugs (AAD) therapy, and catheter ablation.

## 2. Definitions

Despite being a widely discussed topic, terminology in literature is often inconsistent and the terms “asymptomatic AF”, “subclinical AF”, “silent AF”, and “atrial high rate episodes (AHRE)” are used to identify different entities (Table 1). Different studies define “asymptomatic AF” as AF diagnosed incidentally [11] or by ECG in patients reporting no symptoms, or, alternatively, with European Heart Rhythm Association (EHRA) score 1 [6,7,8,9,10,12]. Other studies [6,7,10] consider silent AF and asymptomatic AF as synonyms. The STROKESTOP trial names AF “silent AF” when the diagnosis is established through screening [13]. However, in other experiences [14,15,16] assessing the benefit of AF screening, the arrhythmia diagnosed through a screening program is simply referred to as AF. Conversely, AHRE are defined, with variable temporal cutoffs according to different studies, as atrial tachycardia episodes recorded by the intra-atrial electrode of implanted devices. However, when an intra-atrial electrogram is available, as in the subgroup of patients of the ARTESiA trial [17], the arrhythmia episode has been defined as “subclinical AF” and not as AHRE. Instead, the ASSERT trial refers to AHRE as subclinical AF [18]: in this study the devices were able to detect atrial arrhythmias, but it remains unclear whether the electrograms were finally analyzed to define the episodes or not. These studies, in fact, consider both asymptomatic and symptomatic AF as “clinical” only when diagnosed by surface ECG. For the purpose of this review we consider device detected atrial tachycardia without electrogram documentation as AHRE; subclinical AF refers to AF either diagnosed through screening programs, incidental findings during routine ECG, or after implanted device electrograms analysis; finally, we define asymptomatic AF in patients with a clearly established AF diagnosis, undergoing a defined clinical management and who remain asymptomatic based on physician’s evaluation. According to these definitions, subclinical and asymptomatic AF are a continuum in the diagnostic process and subsequent management of AF: in fact, once the diagnosis has been confirmed and treatment has begun, subclinical AF should be referred to as clinical asymptomatic AF. 

## 3. Pathophysiological Links between AF, Dementia, and Stroke

AF confers an increased risk of cognitive decline and dementia development independently from stroke occurrence. Indeed, this has been recently demonstrated in a large cohort study conducted on 262,611 patients registered in the Korea National Health Insurance Service—Senior, 60 years of age or older and no history of valvular heart disease, stroke, dementia, and AF before enrolment [24]. After adjustment, in the incident AF population the risk of developing dementia was increased after censoring for stroke (HR, 1.27; 95% CI 1.18–1.37). Interestingly, anticoagulation therapy was associated with a lower risk of developing dementia (HR, 0.61; 95% 0.54–0.68). These results are consistent, in fact, with a recent meta-analysis showing a 30% increased risk of AF patients to develop dementia regardless of cerebrovascular events [4].

The association between AF and cognitive impairment in patients without clinical stroke has been linked to several possible mechanisms: micro-embolic events occurring in cerebral circulation may lead to silent cerebral ischemia (SCI), visible by neuroimaging techniques as magnetic resonance imaging (MRI). The correlation between SCIs and cognitive impairment has been demonstrated by ad hoc questionnaires and tests designed to examine cognitive function, both in retrospective [25] and prospective studies [26]. Additionally, a computational analysis showed that AF directly affects cerebral hemodynamic, resulting in hypoperfusions and hypertensive events, possibly associated with non-embolic SCI and microbleedings in the deep cerebral circle [27,28]. Dementia and cognitive decline, however, can also be caused by clinical strokes, a well-known and feared complication of AF. Several questions on the relation between AF and stroke are actually still unsolved: the classical mechanism, based on the Virchow’s triad [29], by which AF causes strokes is thrombus formation in the left atrium (typically in the left atrial appendage), and its subsequent embolism in a cerebral vessel. Nevertheless, studies conducted in patients with implanted devices and who suffered a thromboembolic event showed that, in a large portion of patients, no AF events occurred the months before [30,31]. 

The link between fibrosis and AF is rather complex, since AF causes atrial remodeling and fibrosis itself causes and sustains AF. Animal experimental models demonstrated that sustained AF induces several structural changes in atrial myocytes, including glycogen accumulation, sarcomeres reduction, mitochondrial modification, collagen deposition, and fibrosis [32]. Late gadolinium enhancement MRI studies demonstrated that more severe atrial remodeling with extensive fibrosis is associated with more advanced disease and a higher risk of AF recurrences after catheter ablation [33]. Voltage map during invasive procedures demonstrated that the magnitude of atrial fibrosis is greater in persistent [34] in comparison to paroxysmal AF, and it predicts ablation success [35]. Finally, AF can be a marker of other atrial abnormalities including atrial dilation, left atrial appendage dysfunction, endothelial disease; all these features may cause thromboembolic stroke without an AF episode occurring prior to the cerebrovascular event. Indeed, an “atrial cardiomyopathy” exists, according to which extensive atrial disease can be, at least partially, the cause rather than the consequence of AF [36,37]. Therefore, it has been proposed [30] that AF can cause atrial remodeling and be the manifestation of an atrial disease that increases per se the risk of stroke and thrombi formation, independently from the heart’s rhythm.

Actually, the association between dementia and the different clinical presentation of AF has not been thoroughly analyzed. In fact, no published studies directly analyzed cognitive impairment in patients with asymptomatic AF. Several studies evaluated the relation between subclinical AF and the risk of stroke, focusing on subclinical atrial tachyarrhythmia detected by implanted devices. Indeed, it has been thoroughly demonstrated that evaluation of pacemakers recordings is a reliable method to detect AF [38]. The TRENDS trial [39], in fact, evaluated stroke and systemic thromboembolic events in patients with at least one stroke risk factor (defined by the CHADS2 score) by assessing pacemaker or defibrillator recordings. The study was meant to detect a difference in stroke rate among patients with a high, low burden, or no events of atrial arrhythmias/tachycardia (AHRE). Unfortunately, the study failed to show any significant differences, likely due to underpowering as the event rate was lower than expected. An ancillary analysis of the MOST study, instead, demonstrated that AHRE in patients with sinus node dysfunction conferred an increased risk of stroke, death, and to develop clinical AF [19]. Of note, patients with previously diagnosed supraventricular arrhythmia were not excluded, and a large portion of patients with AHRE presented symptoms. Nevertheless, the authors concluded that symptoms were poorly correlated with and were not reliable markers of AHRE. Analogous results were found in the ASSERT trial, which demonstrated that AHRE in patients without a history of AF confer a greater risk of developing clinical AF and are associated with a higher rate of stroke and systemic thromboembolic events [18].

Finally, also AF burden needs consideration: as thoroughly explained by Kennedy [40], AF burden defines the overall amount of AF duration among each patient, providing additional insight on the magnitude of AF episodes. Many clinical conditions (such as, genetics, obesity, hypertension, diabetes, heart failure, older age [40]) affect AF burden and have an important role in AF morbidity. Indeed, it is now clear that prompt correction of comorbidities and cardiovascular risk factors reduces AF burden [41,42] and is, therefore, recommended in all patients suffering from AF [43,44]. Several arrhythmic entities, including AHRE, detected by non-invasive ECG monitoring and implanted devices, are markers of AF and contribute to define the AF burden [40]: however, despite the association between persistent AF and worse prognosis [45,46,47], current guidelines suggest to base the decision of anticoagulation therapy for stroke prevention on patient’s risk factors (quantified by CHA2DS2-VASc score) and not on AF burden. In fact, an AF burden cutoff identifying an increased risk of stroke or mortality has not yet been established, and its quest is at the heart of many studies.

## 4. Asymptomatic AF and Its Impact on Prognosis

Whether asymptomatic presentation of AF confers a worse prognosis than symptomatic AF is still debated (Table 2). A prospective observational study conducted by Boriani et al. on 3119 patients with AF (the EORP-AF Registry) demonstrated that patients with asymptomatic AF, in comparison to symptomatic patients, have a worse prognosis and an increased risk of mortality at 1 year. At multivariate analysis, however, EHRA score I was not independently associated with a worse prognosis. Therefore, increased mortality was likely mainly driven by the differences in patients’ comorbidities and global risk rather than to the different AF presentation [7]. Another study reported that both patients with subclinical AF and atypical presentations of AF (fatigue, shortness of breath, chest pain, light-headedness, syncope, decreased exercise tolerance without palpitations) are at higher risk of cerebrovascular events (CVE) in comparison with patients with typical AF symptoms (palpitations). Moreover, the risk of cardiovascular and global mortality was greater in asymptomatic patients after adjustment for confounding factors (CHA2DS2-VASc score and age) [11]. A sub-study conducted on the PREFER in AF Registry showed that ischemic stroke and TIA occurred with similar frequency in symptomatic and asymptomatic patients [6]. These results confirm, in fact, those of a retrospective analysis of the AFFIRM trial that revealed no differences in outcomes after adjustment for baseline characteristics between patients with asymptomatic and symptomatic AF [12]. Conversely, in a sub-analysis of the RACE study, asymptomatic AF was associated with improved prognosis (lower hospitalization due to heart failure and antiarrhythmic drugs adverse effect rate) in comparison with symptomatic patients, but no differences were found on the risk of thromboembolic events [10]. However, these trials were not designed to evaluate the prognostic implications of different subtypes of AF, and AF presentation was not considered when deciding treatment throughout follow up. 

Bearing in mind that the first manifestation of AF can be an ischemic stroke with severe consequences (as death, dementia, and lower quality of life), that AF is first diagnosed in 11%–24% of patients with recent ischemic stroke or TIA [48,49], and that AF-associated strokes have a high risk of recurrences, interest in the screening for subclinical AF has grown during the recent years.

## 5. Screening for Atrial Fibrillation

A screening program to early detect subclinical AF entails several questions: what is the appropriate method for the screening? Who should be screened? Is a screening program cost-effective? Current European guidelines for the management of AF suggest opportunistic screening in patients older than 65 years by pulse check or single-lead ECG portable devices (Class of recommendation I). In patients with an implanted device it is recommended to evaluate the occurrence of AHRE on a regular basis: if AHRE are detected, AF must be searched by further electrocardiographic monitoring prior to starting treatment. In patients with cryptogenic ischemic stroke or TIA an initial ECG followed by electrocardiographic monitoring for 72 h is recommended (Class of recommendation I), and a long term strategy should be considered (Class of recommendation IIa) [5]. The AHA/ACC/HRS guidelines, instead, do not provide definite recommendations for screening [50]. In addition, a statement appears suggesting that ECG based screening does not improve detection rate of asymptomatic AF in comparison with a pulse palpation based approach, and there are currently no evidence to support the benefits of an ECG based screening [51,52].

Several methods have been proposed for AF screening in populations with risk factors: pulse palpation has been described as an effective method to detect subclinical AF [14]. However, pulse characteristics are seldom evaluated in primary care settings and adequately trained patients’ compliance in self-monitoring is low in the long term [15]. Handheld ECG is a reliable and effective method for AF screening in at risk populations, with a fourfold increase in AF diagnosis over 12 months according to the REHEARSE-AF study [16]. This study showed that stroke and TIA incidence was not statistically different in screened and unscreened patients. However, clinical events were not considered as a primary outcome, and the study was not designed and powered to detect a significant difference in these events. The ongoing STROKESTOP trial aims at evaluating the efficacy and cost effectiveness in reducing stroke incidence of an AF screening program based on single lead discontinuous ambulatory ECG monitoring in a 75–76 years old population from two regions in Sweden [13]. An analysis of the ongoing investigation demonstrated that this strategy increased AF detection fourfold, as AF has been first diagnosed in 3% of the screened-population; 93% of patients with new detection of AF have initiated oral anticoagulation (OAC) therapy [53]. Awaiting for the conclusion of the trial and its final results, a simulation study based on the study design and available data of this trial, concluded that screening for AF with an ECG recorder is cost effective with a reduction of eight strokes, and an increase of 11 life-years and 12 quality-adjusted life years (QALYs) per 1000 screened patients [54].

In patients who suffered a cryptogenic stroke and in whom standard 24 h ECG monitoring failed to detect AF, two randomized trials, instead, demonstrated the benefits of further prolonging rhythm monitoring. The CRYSTAL-AF trial [55,56] randomized 441 patients, 40 years of age or older, who suffered a cryptogenic stroke or TIA, without history of AF and no other indications to anticoagulant therapy, to implant a cardiac monitoring device or standard care: the AF detection rate was higher in the long term monitoring strategy group both at 6 and 12 months (HR 6.4; 95% CI 1.9–21.7; HR 7.3; 95% CI 2.6–20.8, respectively). The EMBRACE trial [57] compared standard care and 30-days non-invasive ECG monitoring to detect AF in patients over 55 years old, with cryptogenic stroke or TIA within the 6 months prior to randomization. By 90 days after the randomization AF lasting 30 s was detected in significantly more patients in the intervention group (absolute difference 12.9%, 95% CI 8.0–17.6), with a number needed to screen of eight. Moreover, in the AHA/ACC/HRS focused update of AF guidelines recommendation for the use of implantable device for AF detection after cryptogenic stroke has been implemented, according to which a more extensive and thorough long-term cardiac monitoring needs to be considered [44].

## 6. Management of Subclinical and Asymptomatic AF

### 6.1. Clinical Approach to the Patient with Asymptomatic Atrial Fibrillation

Clinical approach to a patient with asymptomatic AF is indeed complex and needs to take into account several aspects of the arrhythmia and presence of comorbidities. First, it must be taken into consideration that asymptomatic AF patients are not always truly asymptomatic, since they may undergo progressive involuntary lifestyle restriction or suffer unrecognized anxiety or depression; moreover, a large portion of AF patients may already present cerebral ischemic lesions (about four out of 10 according to results of the prospective observational SWISS-AF study [58]) which have been demonstrated to affect cognitive functions [26,58]. To assess definitively whether a patient is truly asymptomatic, cardioversion should be performed, and symptoms and quality of life evaluated after sinus rhythm restoration; subsequently, in those who felt improvement after sinus rhythm restoration, a rhythm control strategy should be pursued. It has been demonstrated that catheter ablation for AF significantly improves quality of life and symptoms [59,60]: however subanalysis of these studies demonstrated that, once the patients were divided according to symptoms intensity, the observed benefit was mostly driven by patients with more severe symptoms, whereas those who at baseline were barely symptomatic had little or no improvement after ablation. Therefore, patients’ symptoms (not only typical AF symptoms such as palpitations, but also fatigue and dyspnea) and exercise tolerance should be thoroughly examined and a quality of life questionnaire administered; comorbidities should be evaluated, since they can be a cause of symptoms experienced by the patient, and promptly treated. Control of cardiovascular risk factors, hypertension, smoke habit, sedentary life, and obesity should be also encouraged and recommended, as they worsen symptoms and play a role in disease progression [44]. Finally, a regular cardiological follow-up is recommended, including clinical examination for heart failure signs and echocardiographic control of ejection fraction; the latter, in fact, is a crucial aspect to promptly recognize and address patients with a reduced ejection fraction to rhythm control strategy by catheter ablation in order to prevent further heart failure progression [5] and reduce mortality, as suggested by the recent CASTLE-AF findings [61].

Treatment strategies for prevention of cognitive decline are, instead, debated [62]: in addition to physical exercise and optimal control of cardiovascular risk factors, appropriate anticoagulation therapy is recommended; non-vitamin K antagonist oral anticoagulants (NOAC) should be preferred because of the higher safety profile. Moreover, a rhythm control strategy, including catheter ablation, is indicated; nevertheless, it should be borne in mind that the benefit of restoring sinus rhythm may be partially offset by subclinical cerebral lesions occurring during the procedure [63,64].

### 6.2. Anticoagulation Therapy

The cornerstone of AF therapy for prevention of stroke and systemic thromboembolic events is OAC therapy. The 2016 ESC guidelines recommend to initiate OAC therapy upon evaluation of the individual stroke risk based on the number of CHA2DS2-VASc risk factors; in the absence of contraindications, OAC therapy is recommended when the CHA2DS2-VASc score is ≥2 in male patients or ≥3 in female patients and it should be considered with CHA2DS2-VASc scores 1 or 2 respectively. Whereas vitamin K antagonists are the only recommended anticoagulants for valvular AF patients, i.e., patients with mechanical heart valve or moderate to severe mitral stenosis, NOAC should be preferred in non-valvular AF patients [5]. The net benefit of CHA2DS2-VASc-guided OAC therapy in preventing stroke occurrence and recurrence in AF patients has been thoroughly demonstrated [65,66]. A large meta-analysis showed the superiority of NOAC over VKA with respect to safety and efficacy [67].

As far as OAC therapy in subclinical AF or AHRE is concerned, there is actually no direct evidence that early initiation of OAC treatment in this setting can improve hard clinical endpoints without significantly increasing bleeding risk [51]. It has been proposed that intermittent OAC might provide a better balance between stroke prevention and bleeding risk than uninterrupted OAC therapy. Nevertheless, the IMPACT trial, which evaluated intermittent versus OAC initiated upon standard physician’s indication after detection of AHRE at implanted defibrillator follow-up sessions, failed to show clinical benefit regarding the composite outcome of stroke, systemic embolism, and bleeding events (HR 1.06; 95% CI 0.75–1.51; *p* value: 0.732) [68]. In this perspective, two ongoing clinical trials are designed to assess the feasibility of continuous OAC therapy in patients with subclinical AF or AHRE recorded by implanted devices (Table 3). The randomized, double-blind, multicenter NOAH-AFNET 6 study, including older patients (>65 years old) with at least one additional CHA2DS2-VASc risk factor but without clinical AF nor any other indication to OAC therapy, will evaluate the efficacy of edoxaban compared to aspirin or no antithrombotic therapy in preventing stroke, cardiovascular death, and systemic embolism after AHRE detection by implanted devices’ recordings; the primary safety endpoint will be major bleeding [23]. The ARTESiA trial is a prospective, multicenter, double-blind, randomized controlled trial, which enrolls patients with risk factors for stroke and subclinical AF detected by implanted devices; patients will be randomized to either apixaban or aspirin with a primary composite endpoint of stroke, TIA, and systemic thromboembolic events during an estimated follow-up of 3 years [17].

### 6.3. Anti-Arrhythmic Drug Management

A rhythm control approach in the subset of patients with subclinical and asymptomatic AF is perhaps even more controversial than the previously discussed OAC therapy. The AFFIRM [69,70] and RACE [71] trials demonstrated no advantage on mortality and stroke in pursuing a rhythm control over a rate control strategy in AF patients. Nevertheless, the CASTLE-AF [61] study showed a clear benefit in cardiovascular death and hospitalizations for worsening heart failure in patients with heart failure and paroxysmal or persistent AF treated by catheter ablation. According to current European guidelines, rhythm control therapy is recommended in symptomatic patients presenting with recent onset AF in order to improve quality of life [5]. 

Some considerations are needed in this regard. First, a subanalysis of the AFFIRM [72] study showed that maintaining sinus rhythm confers half the risk of death compared to AF persistence. AAD, however, were associated with increased mortality after adjustment for sinus rhythm restoration, therefore the authors concluded that the beneficial effects of AAD in maintaining sinus rhythm were offset by their adverse effects, resulting in no net survival advantage. Similarly, an observational study [73] comparing stroke and TIA incidence in patients treated with either rate or rhythm control strategy (*n* = 41.193 and 16.325 respectively) showed that rhythm control strategy was associated with a lower risk of stroke and TIA during a mean follow-up of 2.8 years at multivariate analysis (HR 0.80; 95% CI 0.74–0.87). After stratification for CHADS2 score, absolute stroke and TIA incidences were reduced only in patients with CHADS2 score ≥ 2. 

### 6.4. Catheter Ablation

The aforementioned studies compared only AAD with rate control therapy, but AF catheter ablation has now become an effective and widespread option for rhythm control: indeed, ESC guidelines do recommend catheter ablation as a first-line alternative to AAD in symptomatic patients with paroxysmal AF. A large meta-analysis [74] including 1481 patients with AF and 11 randomized controlled trials compared the efficacy and safety of catheter ablation versus AAD therapy (*n* = 785 and 696 respectively): catheter ablation was associated with lower AF recurrences (RR, 0.40; 95% CI 0.31−0.52; *p* value = 0.00001) both as first- and second-line approaches (RR, 0.52; 95% CI, 0.30−0.91; *p* value = 0.02 and RR, 0.37; 95% CI, 0.29−0.48; *p* value < 0.00001, respectively), but there was a significant increase of adverse events incidence (RR, 2.04; 95% CI, 1.10–3.77; *p* value = 0.02). However, after stratification by date, no difference in safety endpoints was found considering only the results of the studies conducted after 2009 (RR, 1.51; 95% CI, 0.55–4,15; *p* value = 0.42). This finding may be due to increased experience and improved technology in catheter ablation, leading to lower complications and adverse events.

Since catheter ablation proved more effective than AAD therapy in maintaining sinus rhythm [74], it has been hypothesized that indication to this procedure could reflect into better long-term prognosis. However, the recent CABANA trial [75], which was supposed to shed light on this clinical dilemma, failed to demonstrate a statistically significant superiority of catheter ablation over both rhythm and rate control pharmacological therapy for a composite primary endpoint including death, disabling stroke, serious bleeding, or cardiac arrest (HR, 0.86; 95% CI 0.65–1.15; *p* value = 0.30). A detailed analysis of the results, however, shows that the study was characterized by a greater than expected cross-over between treatment groups, with 301 (27.5%) patients assigned to medical therapy switching to catheter ablation. Indeed, in the 12-month per-protocol analysis of the results, patients who had undergone catheter ablation presented a reduced risk of meeting the primary endpoint than those managed pharmacologically (HR 0.73; 95% CI 0.54–0.99).

Several other investigations, albeit limited by their unrandomized nature, provided evidence in favor of catheter ablation. One study comparing patients with similar CHADS2 scores showed that catheter ablation confers a risk of ischemic stroke similar to that of the population without AF, but the details on OAC treatment were lacking [76]. A multicenter study conducted by Hunter et al. [77] compared patients undergoing AF ablation with a cohort treated with medical therapy and an AF-free cohort representing the general population; of note, in the medical therapy cohort CHADS2 score was higher than in catheter ablation group (1.6 ± 1.2 and 0.7 ± 0.9 respectively). After a mean follow-up of 3.1 years, catheter ablation was associated with a lower risk of stroke and death in comparison with medical therapy, presenting a stroke rate comparable to the general population. Moreover, freedom from AF was a protective factor against stroke at multivariate analysis (HR 0.33; 95% CI 0.17–0.67). Discontinuation of OAC therapy occurred in 64% of patients who underwent pulmonary veins (isolation (85% of these were on single antiplatelet agent therapy) and it was more frequent in patients without AF recurrences in comparison to patients with AF recurrences, despite a small difference in CHADS2 score between the two groups (0.7 ± 0.9 vs. 0.9 ± 0.9, respectively). These findings confirm those of another observational study [78] which reported no statistically significant difference between patients who stopped or continued OAC after AF ablation. Along with history of stroke, and unlike OAC interruption, except in intermediate risk patients, recurrent AF was a predictor of thromboembolic events, whereas OAC continuation was associated with an increased risk of bleeding. Another multicenter observational study [79] recruiting 1500 AF patients compared rate control and OAC therapy with catheter ablation associated with either OAC continuation or discontinuation after the procedure: no differences were found as for thromboembolic events incidence (2.2% in rate control strategy and OAC, 1% in catheter ablation with OAC, 1.4% catheter ablation and OAC discontinuation, *p* value = 0.45), whereas OAC discontinuation after catheter ablation conferred a lower risk of hemorrhagic events (2.4% in rate control strategy and OAC, 1.8% in catheter ablation with OAC, and no events in catheter ablation and OAC discontinuation, *p* value < 0.001). 

**Figure 1:**
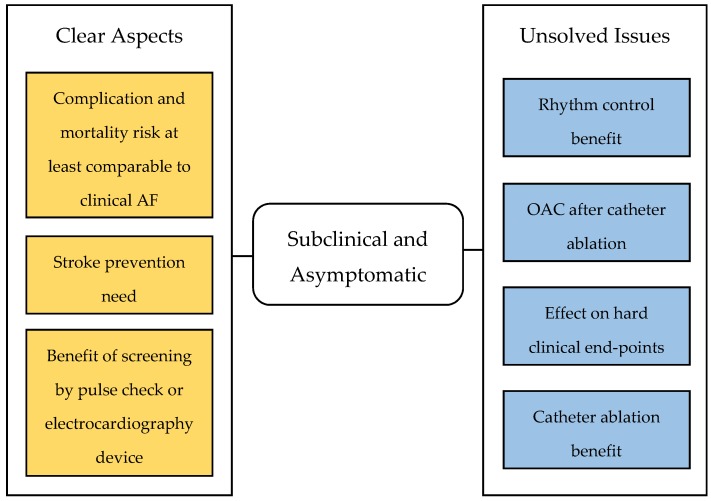
Current certainties and doubts on the management of subclinical and asymptomatic AF.

Based on the aforementioned studies, preservation of sinus rhythm, achieved by AAD therapy or catheter ablation, holds the potential to confer an improved clinical outcome in the general population. Among patients with fewer risk factors and lower event rates than those with heart failure, a longer follow-up and greater sample sizes are possibly needed to identify a statistically significant benefit in hard clinical end-points. Indeed, maintenance of sinus rhythm can prevent atrial structural remodeling process accompanying the arrhythmia, hampering disease progression. In this perspective, the currently ongoing EAST [80] trial will assess if rhythm control therapy in the early phase following AF diagnosis can improve prognosis in comparison with a more conservative approach; the trial primary endpoint is a composite of cardiovascular death, stroke, worsening heart failure and myocardial infarction; cognitive function will be evaluated as a secondary outcome as in the STROKESTOP, NOAH-AFNET 6, and OCEAN studies. Additionally, two ongoing trials will shed light on another quandary in clinical management of AF patients: the open, multicenter, randomized OCEAN trial [81] will compare rivaroxaban and acetylsalicylic acid efficacy in reducing stroke, systemic embolism and subclinical brain lesions incidence among high-risk patients free from AF for at least 1 year after pulmonary veins isolation after a 3 years follow-up period. In the OAT trial (NCT01959425) patients without AF recurrences three months after catheter ablation will be randomized to suspend or continue OAC. Should these trials meet their outcomes, a rhythm control strategy with catheter ablation in an early phase of the disease would be recommended aiming to interrupt disease progression and prevent AF-related complications. Moreover, it would be possible to consider catheter ablation in asymptomatic patients as well; as a matter of fact, as thoroughly explained by Kalman et al. [82], subclinical AF can become symptomatic with arrhythmia progression, therefore it may prove useful to treat the arrhythmia in this early “window of opportunity” in order to prevent symptoms onset and atrial remodeling. Finally, patients’ compliance to OAC therapy for AF diagnosed after a single ECG in the absence of any symptoms is of great concern in clinical practice; in this scenario catheter ablation may be a valid tool potentially providing the expected benefits (Figure 1).

## 7. Conclusions

The treatment of asymptomatic AF patients should be personalized, evaluating individual risk factors and comorbidities as well as patient’s own preference. High risk individuals with heart failure, should be promptly treated, and catheter ablation should be offered due to the strong evidence supporting AF ablation in this setting. As for patients with early-diagnosed asymptomatic AF and no comorbidities, ongoing clinical trials will assess the benefits of catheter ablation and pharmacological rhythm control therapy, but currently there is not enough evidence to clearly support this approach.

## Figures and Tables

**Table 1:** Details on the terminology used in available literature.

Authors (Study Year)	AHRE/Subclinical/Asymptomatic AF Diagnostic Method	Terminology Used in the Study	Terminology Used in This Review
Implanted device monitoring			
Glotzer et al. (2003) [19]	AHRE: atrial rate ≥ 220 bpm lasting at least 5 min (detected by pacemaker)	AHRE	AHRE
Glotzer et al. (2006) [20]	AHRE: atrial rate > 175 bpm lasting at least 20 s	Device-detected atrial tachycardia (AT)/AF burden (AHRE)	
Hohnloser et al. (2006) [21]	AHRE: atrial rate ≥ 190 bpm lasting at least 6 min (detected by pacemaker or ICD)	Asymptomatic AF/AHRE	
Ip et al. (2009) [22]	AHRE: atrial rate ≥ 220 beat/min lasting at least 5 min,	AHRE	
Kirchhof et al. (2017) [23]	AHRE: atrial rate ≥ 180 bpm lasting at least 6 min	AHRE	
Lopes et al. (2017) [17]	One episode of device-detected subclinical AF lasting at least 6 min. Subclinical AF requires at least one episode of electrogram confirmation	Subclinical AF	Subclinical AF
Non-Invasive ECG monitoring			
Flaker et al. (2005) [12]	AF diagnosed with ECG or rhythm strip. Symptoms evaluated by a questionnaire	Asymptomatic AF	Asymptomatic AF
Rienstra et al. (2014) [10]	Recurrent persistent AF without symptoms according to a questionnaire	Asymptomatic AF/Silent AF	
Boriani et al. (2015) [7]	ECG diagnosed AF and EHRA score I	Asymptomatic AF/Silent AF	
Freeman et al. (2015) [9]	Electrocardiographically documented AF and EHRA score I	Asymptomatic AF	
Bakhai et al. (2016) [6]	ECG diagnosed AF and EHRA score I	Asymptomatic AF/Silent AF	
Siontis et al. (2016) [11]	AF detected incidentally (routine physical examination, preoperative evaluation, emergency department or clinic visit for unrelated problem)	Asymptomatic AF	Subclinical AF
Jaakkola et al. (2017) [15]	ECG diagnosis in a screening program	AF	
Halcox et al. (2017) [16]	Device detected AF in a screening program	AF	
Friberg et al. (2013) [13]	Device detected AF in a screening program and confirmed by Holter for uncertain cases	Silent AF	

AF: atrial fibrillation; AHRE: atrial high rate episodes; ICD: implantable cardioverter-defibrillator.

**Table 2:** Summary of studies on the clinical relevance of asymptomatic and subclinical atrial fibrillation (AF).

Authors (Study Year)	AHRE/Subclinical/Asymptomatic AF Diagnostic Method	Stroke and TE Incidence (%) *	Mortality (%) *	Correlation with Stroke or Mortality
Implanted device monitoring				
Glotzer et al. (2003) [19]	AHRE: atrial rate ≥ 220 bpm lasting at least 5 min (detected by pacemaker)	AHRE: 33/160 (20.6) No AHRE: 16/152 (10.5)	AHRE: 28/160 (17.5) No AHRE: 16/152 (10.5)	Yes (for total mortality, stroke, and AF development)
Glotzer et al. (2009) [39]	AHRE: atrial rate > 175 bpm lasting at least 20 s. Patients were stratified according to 30 days window monitoring in: zero, low, and high AHRE burden	Annualized TE incidence rate: zero AHRE burden 1.1%; Low AHRE burden 1.1%; High AHRE burden 2.4%	NA	No
Healey et al. (2012) [18]	AHRE: atrial rate ≥ 190 bpm lasting at least 6 min (detected by pacemaker or ICD)	AHRE in the previous 3 months: 11/261 (4.2) No AHRE in the previous 3 months: 40/2319 (1.7)	From vascular causes AHRE in the previous 3 months: 19/261 (7.3) No AHRE in the previous 3 months: 153/2319 (6.6)	Yes
Non-Invasive ECG monitoring				
Flaker et al. (2005) [12]	AF diagnosed with ECG or rhythm strip. Symptoms evaluated by a questionnaire	Asymptomatic AF: 21 Symptomatic AF: 136	Asymptomatic AF: 60 (19) Symptomatic AF: 606 (27)	No (in comparison with symptomatic patients)
Rienstra et al. (2014) [10]	Recurrent persistent AF without symptoms according to a questionnaire	Asymptomatic AF: 8/157 Symptomatic AF: 28/365	Asymptomatic AF: 9/157 Symptomatic AF: 26/365 (death from cardiovascular causes)	No †
Boriani et al. (2015) [7]	ECG diagnosed AF and EHRA score I	EHRA I: 10/962 (1.0%) EHRA II–IV: 15/1344 (1.1%)	EHRA I: 102/1086 (9.4%) EHRA II–IV: 65/1556 (4.2%)	Yes (for mortality compared to symptomatic patients)
Freeman et al. (2015) [9]	Electrocardiographically documented AF and EHRA score I	EHRA I: 99/3682 EHRA II–IV: 168/5918	EHRA I: 311/3682 EHRA II–IV: 561/5918	No ‡
Bakhai et al. (2016) [6]	ECG diagnosed AF and EHRA score I	EHRA I: ischemic stroke 8/489 (1.6) TIA 7/488 (1.4) arterial embolism 2/488 (0.4) EHRA II–IV: ischemic stroke 44/5514 (0.8) TIA 73/5510 (1.3) arterial embolism 11/5514 (0.2)	NA	No (only EHRA score IV was associated with a higher events occurrence)
Siontis et al. (2016) [11]	AF detected incidentally (routine physical examination, preoperative evaluation, emergency department, or clinic visit for unrelated problem)	HR compared to typical AF: Subclinical AF 2.60 (95% C.I. 1.10–6.11) Atypical AF 3.12 (95% C.I. 1.27–7.66)	HR compared to typical AF: Subclinical AF 4.01 (95% C.I. 2.32–6.91) Atypical AF 3.19 (95% C.I. 1.78–5.71)	Yes (compared to typical AF)

AF: atrial fibrillation; AHRE: atrial high rate episodes; NA: not applicable; TIA: transient ischemic attack; TE: thromboembolism * If not otherwise indicated. † In this study asymptomatic AF compared to symptomatic presentation conferred a lower risk of heart failure and severe antiarrhythmic drugs adverse effects. ‡ In this study AF symptoms and decreased quality of life were associated with higher risk of hospitalization.

**Table 3:** Details of ongoing trials.

Name of Study	Number of Patients	Study Arms	Primary Endpoints	Secondary Endpoints
STROKESTOP (NCT01593553)	7173 (in screening) 14,381 (controls not screened)	Intervention group: ECG screening for AF using intermittent ECG recorder. Control group: standard of care	Composite of ischemic and hemorrhagic stroke, systemic embolism, major bleeding requiring hospitalization, and all-cause mortality	Each single component of the composite primary outcome; dementia; cardiovascular mortality; hospitalization due to cardiovascular disease; cost-effectiveness; OAC initiation and compliance; AF detection; pulmonary embolism and deep vein thrombosis
NOAH-AFNET 6 (NCT02618577)	2686 (3400 estimated) patients (≥65 years old and ≥1 additional CHA2DS2-VASc factor) with AHRE documented by implanted devices	Intervention group: Edoxaban (standard AF dosing) Control group: ASA or placebo	Composite of stroke, systemic embolism and cardiovascular death (measured as time from randomization to event occurrence)	MACE (cardiac death, MI, acute coronary syndrome), all-cause death, major bleeding events, quality of life changes at 12 and 24 months, patient satisfaction at 12 and 24 months, cost effectiveness and health resource utilization, autonomy status changes in patients affected by stroke during study participation, cognitive function at 12 and 24 months
ARTESiA (NCT01938248)	≈4000 patients with subclinical AF at high risk for stroke (estimated)	Intervention group: Apixaban (standard AF dosing) Control group: ASA (81 mg/die)	Efficacy outcome: composite of stroke (including TIA) and systemic embolism. Safety outcome: major bleeding	Ischemic stroke; MI; vascular death; total death; composite of stroke, MI, systemic embolism and total death; composite of stroke, MI, systemic embolism, total death, and major bleeding.
EAST (NCT01288352)	2789 patients with new AF (<1 year) and risk factors for stroke	Intervention group: guidelines-based therapy andearly rhythm control therapy (AAD or PVI) Control group: usual care	First coprimary outcome: composite of cardiovascular death, stroke (including TIA), acute coronary syndrome, and worsening of heart failure. Second coprimary outcome: nights in hospital per year	Cardiovascular death, stroke, worsening of heart failure, acute coronary syndrome, time to recurrent AF, cardiovascular hospitalizations, all-cause hospitalizations, left ventricular function, quality of life, cognitive function
OCEAN (NCT02168829)	1572 patients free from AF for at least 1 year after catheter ablation for non-valvular AF (estimated)	Active Comparator: rivaroxaban 15 mg/die Active Comparator: ASA 75–160 mg/die	Composite of clinically overt stroke, systemic embolism, and covert stroke detected by brain MRI	Each single component of the composite primary outcome; major bleeding, clinically relevant non-major bleeding, minor bleeding and their composite; overt intracranial hemorrhage; microbleedings as detected by MRI; TIA; all-cause mortality; net clinical benefit; occurrence of nonprimary end point MRI changes; correlation of AF burden/recurrence to occurrence of clinical or covert stroke; neuropsychological testing; quality of life
OAT (NCT01959425)	100 patients free from AF for at least 3 months after catheter ablation and at high risk for stroke (estimated)	Intervention group: OAC discontinuation Control group: OAC continuation	Composite of any major thromboembolic event and major hemorrhagic complication	Bleeding; hospitalization; mortality; quality of Life; AF recurrence; repeat ablation
SWISS-AF (NCT02105844)	2415 AF patients	NA	Stroke or systemic embolism	Hospitalization for heart failure

AAD: antiarrhythmic drugs; AF: atrial fibrillation; AHRE: atrial high rate episodes; ASA: acetylsalicylic acid; MACE: major adverse cardiovascular events; MI: myocardial infarction; MRI: magnetic resonance imaging; NA: not applicable; OAC: oral anticoagulant; PVI: pulmonary veins isolation; TIA: transient ischemic attack.
